# Anaemia and its association with iron, vitamin B12 and folate deficiency in school-age children and adolescents: a cross-sectional study of primary-care laboratory records (2019–2025)

**DOI:** 10.1136/bmjpo-2026-005163

**Published:** 2026-09-02

**Authors:** Kevser Erdoğan, Hülya Doğan Tiryaki, Osman Kurt, Nefise Şeker, Osman Küçükkelepçe

**Affiliations:** 1 Department of Public Health, Antalya Training and Research Hospital, Antalya, Turkey; 2 Department of Public Health, Faculty of Medicine, Gaziantep University, Gaziantep, Turkey; 3 Department of Public Health, İnönü University Faculty of Medicine, Malatya, Turkey; 4 Department of Public Health, Şanlıurfa Provincial Health Directorate, Şanlıurfa, Turkey; 5 Department of Public Health, Gülhane Faculty of Medicine, University of Health Sciences, Ankara, Turkey

**Keywords:** Child Health, Epidemiology, Cross-Sectional Studies, Adolescent Health

## Abstract

**Objective:**

To determine the frequency of anaemia and its association with iron, vitamin B_12_ and folate deficiency in school-age children and adolescents.

**Design:**

Cross-sectional study.

**Setting:**

Family health centres (primary care) in Antalya, Türkiye, 2019–2025.

**Participants:**

Children aged 6–18 years with a complete blood count; the first eligible record per child was analysed (n=240 613).

**Main outcome measures:**

Anaemia, defined by WHO age-specific and sex-specific haemoglobin thresholds; iron (ferritin <15 µg/L or transferrin saturation <16%), vitamin B_12_ (<200 pg/mL) and folate (<3 ng/mL) deficiency; associations assessed by χ² test, ORs and multivariable logistic regression. Because total iron-binding capacity was not measured directly, transferrin saturation was derived as serum iron divided by the sum of serum iron and unsaturated iron-binding capacity, expressed as a percentage; iron deficiency was defined as ferritin <15 µg/L or transferrin saturation <16%, whichever was available.

**Results:**

Anaemia prevalence was 9.5% (95% CI 9.4% to 9.6%), higher in girls (13.6%) than boys (4.2%) and rising with age (6–11 years 4.8%; 15–18 years 12.9%; both p<0.001). Most anaemia was microcytic (76.0%). Iron deficiency was present in 50.4%, vitamin B_12_ deficiency in 53.7% and folate deficiency in 0.8%. In multivariable analysis, iron deficiency (adjusted OR 4.15, 95% CI 3.99 to 4.31), female sex and older age were independently associated with anaemia (all p<0.001).

**Conclusions:**

Anaemia remains common and largely preventable in school-age children and adolescents, with iron deficiency as the principal correlate. Adolescent girls are the priority group for iron-status screening and intervention.

**WHAT IS ALREADY KNOWN ON THIS TOPIC**

- Anaemia is a major global health problem in which school-age children and adolescents—particularly girls—are a priority risk group with iron deficiency as the leading cause, yet large real-world estimates of anaemia and its micronutrient correlates in this age group from Türkiye are scarce.

**WHAT THIS STUDY ADDS**

- In 240 613 children from 7 years of primary-care laboratory records, anaemia prevalence was 9.5%, was predominantly microcytic and rose steeply among adolescent girls.
- Iron deficiency was the strongest independent correlate of anaemia, supporting prioritisation of adolescent girls for iron-status screening in primary care.
- The joint measurement of iron, vitamin B_12_ and folate showed that combined deficiency—most often iron together with vitamin B_12_—was the most common deficiency pattern, and that about half of anaemic children with a normal mean corpuscular volume nonetheless had two or more deficiencies, so red-cell size alone should not gate micronutrient testing.

**HOW THIS STUDY MIGHT AFFECT RESEARCH, PRACTICE OR POLICY**

- A normal mean corpuscular volume does not exclude treatable combined micronutrient deficiency in an anaemic child, so red-cell size should not be used to decide whether to test for iron, vitamin B_12_ and folate in primary care.

## Introduction

 Anaemia is the most common haematological disorder worldwide and a substantial public-health burden. In 2021, the global prevalence of anaemia across all ages was 24.3%, corresponding to 1.92 billion prevalent cases.^1^ The burden falls disproportionately on children and women in low- and middle-income settings, and dietary iron deficiency is consistently identified as the single leading cause of anaemia globally.^2–4^

School-age children and adolescents are a priority risk group because rapid growth, rising iron requirements and—particularly in adolescent girls—menstrual losses increase their susceptibility to iron depletion and anaemia.^5 6^ Anaemia and iron deficiency during these years are associated with impaired cognition, attention and academic performance, and with partly irreversible neurodevelopmental deficits, which makes timely detection important.^7 8^

The aetiology of anaemia is multifactorial. Beyond iron deficiency, vitamin B_12_ and folate deficiency are correctable causes that can produce megaloblastic or dimorphic anaemia.^9 10^ Iron-deficiency anaemia is typically microcytic, and ferritin and transferrin saturation are key markers of iron status.^11–14^ Vitamin B_12_ deficiency is reported at high frequency among children and adolescents where the intake of animal-source foods is limited, and may carry neurocognitive consequences even without overt megaloblastic anaemia, whereas folate status varies markedly with fortification policy and diet.^9 10 15^

Despite the scale of the problem, large, real-world estimates of anaemia and its micronutrient correlates in Turkish school-age children and adolescents are scarce, and most available studies are relatively small, single-setting samples.^16 17^ Primary-care laboratory records offer a population-representative source for estimating anaemia and associated biochemical deficiencies at scale. The present study period also spans the COVID-19 pandemic, during which the use of health services and routine laboratory testing was substantially disrupted.^18^

Using 7 years of primary-care laboratory records, we aimed to determine the frequency of anaemia in children and adolescents aged 6–18 years; to describe its distribution by sex, age and calendar period; and to quantify its association with iron, vitamin B_12_ and folate deficiency.

## Methods

### Study design and population

This cross-sectional study used the laboratory records of children and adolescents who attended family health centres in Antalya, Türkiye, and whose samples were analysed at the provincial public-health laboratory between January 2019 and December 2025. Individuals aged 6–18 years with an available complete blood count were included. In line with the age range of compulsory schooling in Türkiye and with the WHO age/sex-specific haemoglobin bands, school age was defined as 6–18 years.^19^

### Variables and definitions

Anaemia was defined using WHO age-specific and sex-specific haemoglobin thresholds: <115 g/L for ages 6–11 years, <120 g/L for ages 12–14 years, and for ages 15–18 years, <120 g/L in girls and <130 g/L in boys.^19^ Anaemia severity (mild, moderate, severe) was classified according to the same source. Erythrocyte morphology was grouped by mean corpuscular volume (MCV) as microcytic (<80 fL), normocytic (80–100 fL) or macrocytic (>100 fL). Iron deficiency was defined as ferritin <15 µg/L or transferrin saturation <16%, with transferrin saturation derived from serum iron and iron-binding capacity.^14^ Vitamin B_12_ deficiency was defined as <200 pg/mL and folate deficiency as <3 ng/mL.^9 15^

Calendar period was classified from the record date, using WHO milestones for the COVID-19 pandemic, into prepandemic (up to 29 February 2020), pandemic (11 March 2020 to 5 May 2023, from the declaration of the pandemic to the termination of the Public Health Emergency of International Concern) and postpandemic (from 6 May 2023 onward).

### Data source, integration and quality control

Monthly laboratory files were merged; personal identifiers were removed and repeat attendances were linked through a de-identified person key. Physiological plausibility limits were applied to all analytes, and out-of-range values (recording/unit errors) were excluded. Consistent with the cross-sectional design, the first eligible record per child was selected as the unit of analysis. From 426 279 records of children aged 6–18 years with a complete blood count, this yielded 240 613 unique children.

### Statistical analysis

Analyses were performed with IBM SPSS Statistics V.22. Non-normally distributed laboratory variables were summarised as median and IQR and categorical variables as counts and percentages; a 95% CI was calculated for the anaemia prevalence. Categorical comparisons used the χ² test. The association between anaemia and iron, vitamin B_12_ and folate deficiency was expressed as odds ratios (OR) with 95% CI. Factors independently associated with anaemia were examined with multivariable logistic regression including sex, age group, iron deficiency and vitamin B_12_ deficiency; adjusted ORs are reported with 95% CI. A two-sided p<0.05 was considered statistically significant. The study is reported in accordance with the Strengthening the Reporting of Observational Studies in Epidemiology (STROBE) statement.

Ferritin, transferrin saturation, vitamin B_12_ and folate were measured only when clinically requested, so the number of children contributing to each analysis differed and was smallest for folate. To avoid excluding the large share of children without a folate result, and because folate deficiency was rare, the multivariable model was restricted to variables available in the great majority of children (sex, age, iron and vitamin B_12_ status); folate was examined in univariable analysis only. The extent and potential consequences of this differential testing are considered in the Discussion.

Because a single record per child was analysed, comparisons across calendar years or pandemic periods contrast different children tested at different times rather than the same individuals followed longitudinally; period figures are therefore descriptive and should not be read as within-child temporal change.

### Patient and public involvement

Patients or the public were not involved in the design, conduct, reporting or dissemination plans of this research, which used de-identified retrospective laboratory records.

## Results

### Characteristics of the study population

A total of 240 613 children and adolescents were analysed. The mean age was 13.2 (3.6) years, and 135 805 (56.4%) were girls. Sociodemographic, haematological and biochemical characteristics are shown in table 1.

**Table 1:** Characteristics of the study population (n=240 613)

Characteristic	Value
Age, years, mean (SD)	13.2 (3.6)
Sex, n (%)	
Girls	135 805 (56.4)
Boys	104 808 (43.6)
Age group, n (%)	
6–11 years	76 793 (31.9)
12–14 years	59 682 (24.8)
15–18 years	104 138 (43.3)
Haematological, median (IQR)	
Haemoglobin, g/L	133 (126–142)
MCV, fL	82.8 (79.2–86.2)
MCH, pg	27.8 (26.4–29.1)
RDW, %	13.7 (13.1–14.4)
Biochemical, median (IQR)	
Ferritin, µg/L	19.0 (10.9–31.1)
Serum iron, µg/dL	75.0 (50.0–103.0)
Transferrin saturation, %	19.7 (12.9–27.5)
Vitamin B_12_, pg/mL	191.0 (140.0–262.0)
Folate, ng/mL	7.8 (6.0–10.4)

MCV, mean corpuscular volume; MCH, mean corpuscular haemoglobin; RDW, red-cell distribution width.

### Frequency and distribution of anaemia

**Figure 1:**
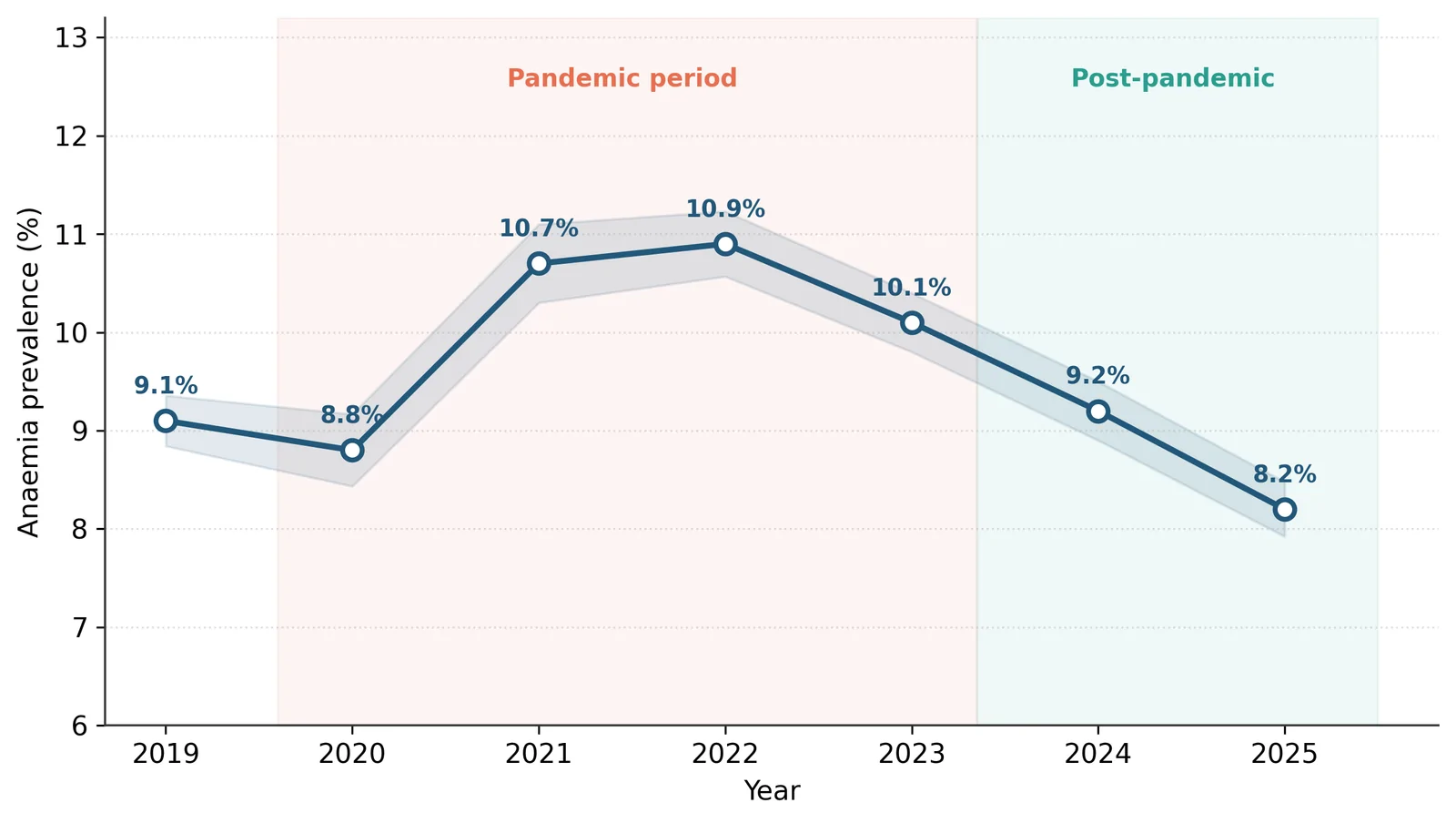
Temporal trend of anaemia prevalence, 2019–2025. The line shows the yearly prevalence with its 95% CI (shaded band); background shading distinguishes the pandemic and postpandemic periods.

**Figure 2:**
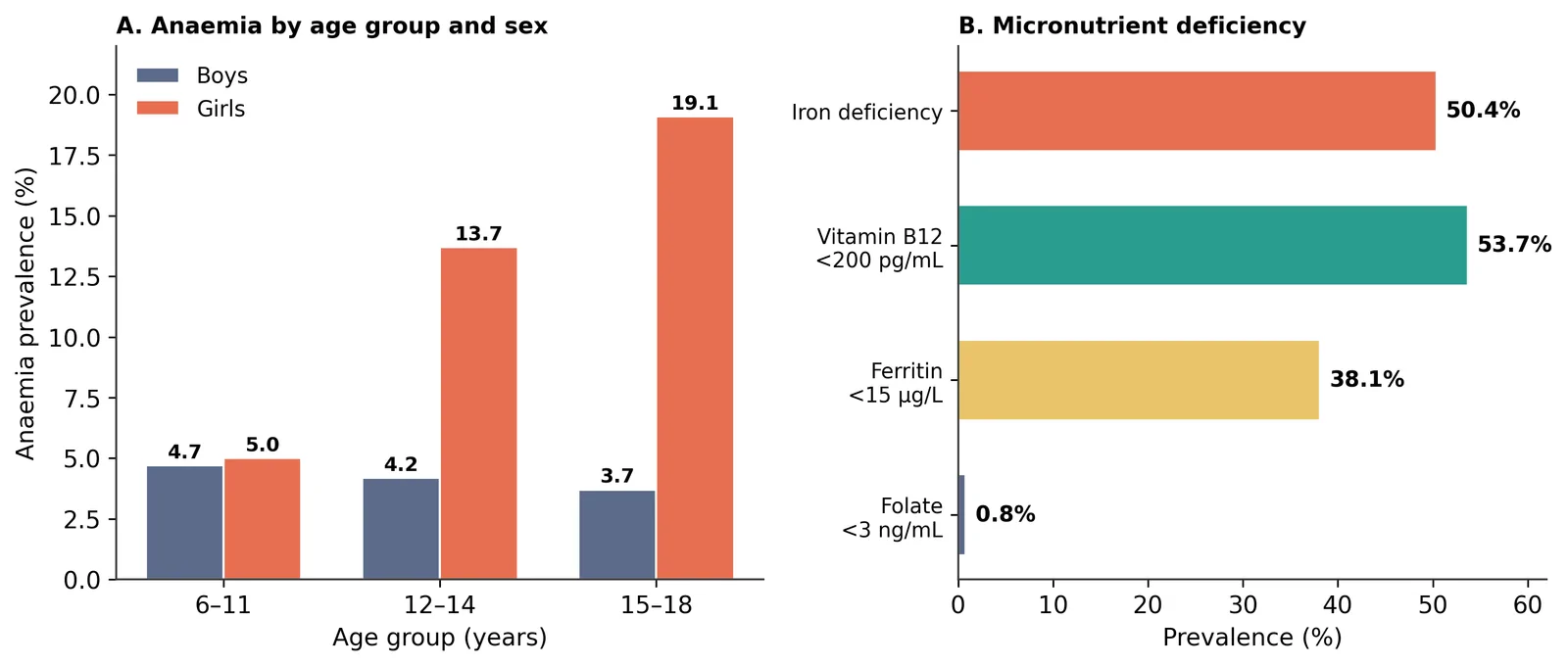
(**A**) Anaemia prevalence by age group and sex. (**B**) Prevalence of micronutrient deficiencies (iron deficiency: ferritin <15 µg/L or transferrin saturation <16%; vitamin B_12_ <200 pg/mL; folate <3 ng/mL).

Anaemia was identified in 22 885 of 240 566 children with an evaluable haemoglobin value, a prevalence of 9.5% (95% CI 9.4% to 9.6%). Among anaemic children, 62.8% had mild, 34.6% moderate and 2.5% severe anaemia and 76.0% were microcytic. Anaemia was more frequent in girls than boys, increased across age groups and differed by pandemic period (all p<0.001; online supplemental table 1, figures 1 and 2).

### Micronutrient deficiencies and their association with anaemia

Iron deficiency was present in 50.4%, ferritin deficiency (<15 µg/L) in 38.1%, vitamin B_12_ deficiency in 53.7% and folate deficiency in 0.8% of children with the corresponding measurement (figure 2). Iron, vitamin B_12_ and folate deficiency were each associated with anaemia, most strongly iron deficiency (OR 4.98, 95% CI 4.8 to 5.16; p<0.001); anaemia occurred in 15.4% of iron-deficient versus 3.5% of iron-replete children (table 2).

**Table 2:** Prevalence of micronutrient deficiencies and their association with anaemia

Deficiency	Prevalence n/N (%)	Anaemia if deficient, %	Anaemia if replete, %	OR (95% CI)	P value
Iron deficiency	109 265/216 803 (50.4)	15.4	3.5	4.98 (4.8 to 5.16)	<0.001
Vitamin B_12_ deficiency	114 556/213 341 (53.7)	10.3	8.5	1.23 (1.2 to 1.27)	<0.001
Folate deficiency	803/94 526 (0.8)	15.4	9.5	1.73 (1.43 to 2.1)	<0.001

Comparisons used the χ² test. Iron deficiency: ferritin <15 µg/L or transferrin saturation <16%.

### Factors independently associated with anaemia

**Figure 3:**
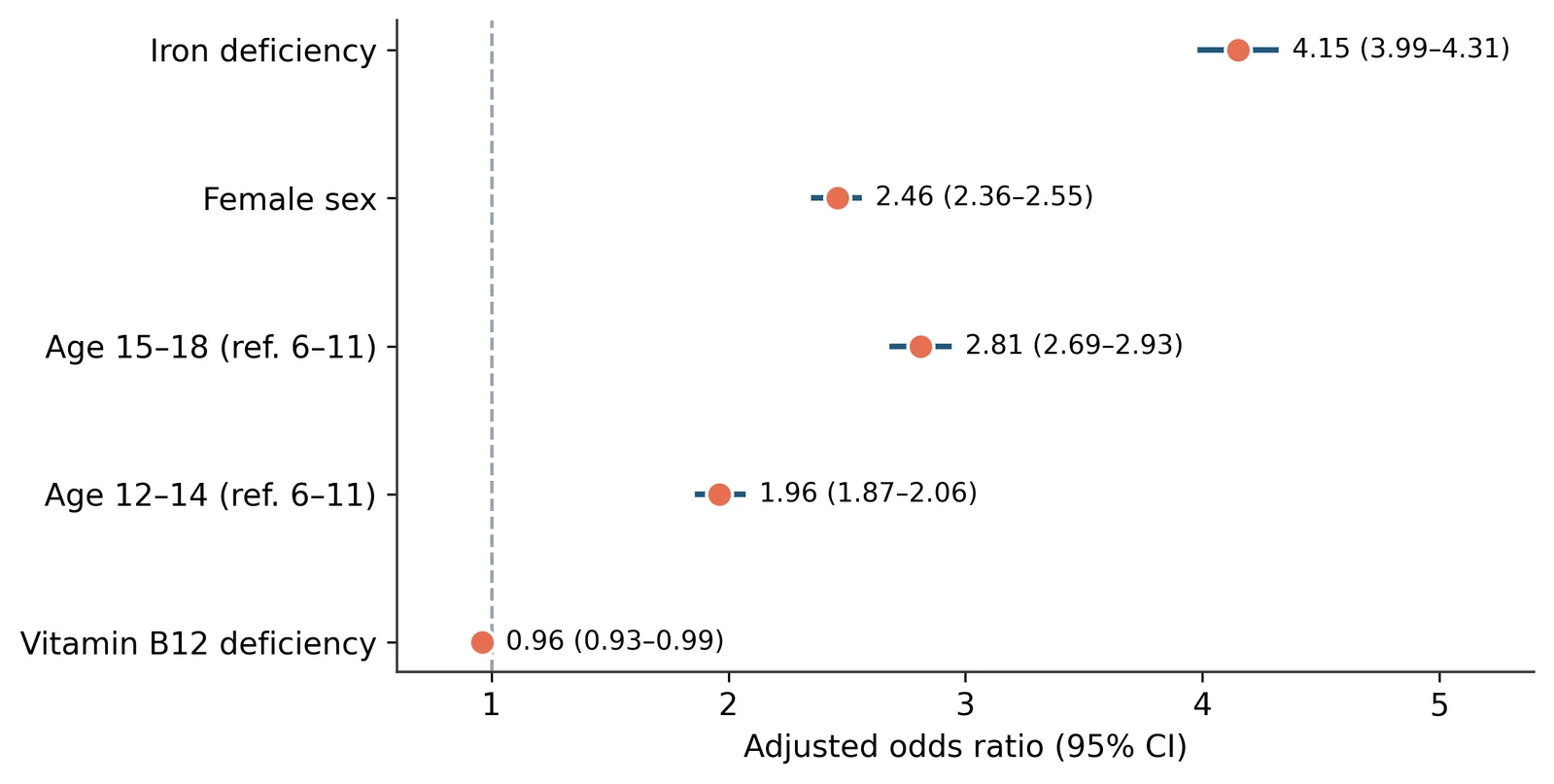
Adjusted ORs with 95% CIs for factors associated with anaemia (multivariable logistic regression). The dashed line indicates the null value (OR=1).

In the multivariable model (n=2 07 601), iron deficiency, female sex and older age were independently and positively associated with anaemia (p<0.001). Iron deficiency carried the highest adjusted odds (adjusted OR 4.15, 95% CI 3.99 to 4.31). After adjustment, vitamin B_12_ deficiency was not positively associated with anaemia (adjusted OR 0.96, 95% CI 0.93 to 0.99; table 3, figure 3).

**Table 3:** Multivariable logistic regression for factors associated with anaemia (n=207 601)

Variable	Adjusted OR (95% CI)	P value
Female sex (ref. male)	2.46 (2.36 to 2.55)	<0.001
Age 12–14 years (ref. 6–11)	1.96 (1.87 to 2.06)	<0.001
Age 15–18 years (ref. 6–11)	2.81 (2.69 to 2.93)	<0.001
Iron deficiency (ref. none)	4.15 (3.99 to 4.31)	<0.001
Vitamin B_12_ deficiency (ref. none)	0.96 (0.93 to 0.99)	<0.001

ref., reference category.

### Co-occurrence of deficiencies and red-cell size

**Figure 4:**
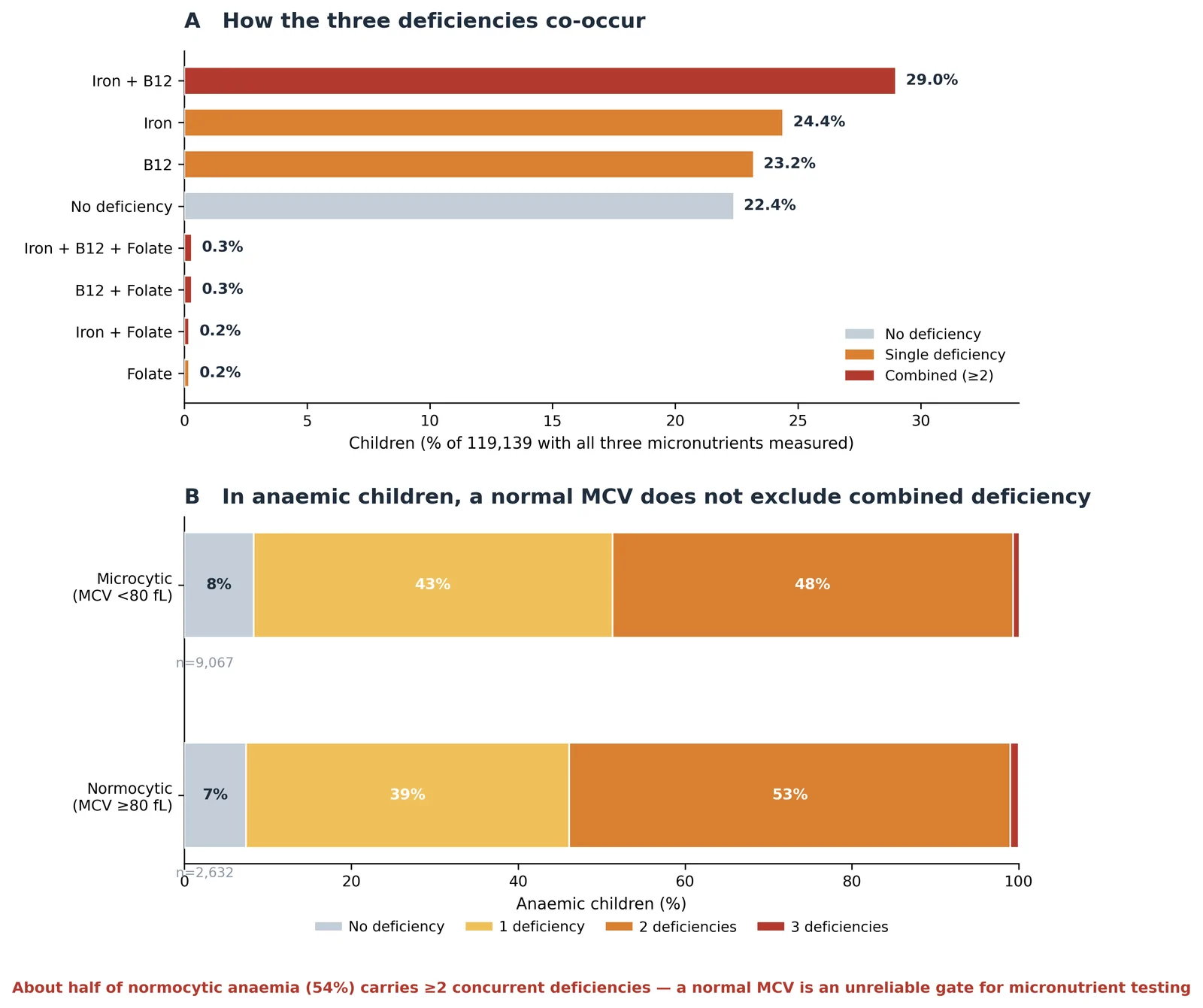
Co-occurrence of iron, vitamin B_12_ and folate deficiency and its relation to red-cell size. (A) The distribution of single and combined deficiencies among the 119 139 children in whom all three micronutrients were measured, with the anaemia prevalence within each group. (B) The number of concurrent deficiencies by mean corpuscular volume category among anaemic children; about half of normocytic anaemia was associated with two or more deficiencies. MCV, mean corpuscular volume.

Because iron, vitamin B_12_ and folate status were assessed together in 119 139 children, we examined how the three deficiencies co-occurred and how this related to red-cell size (table 4, figure 4). Isolated deficiencies were common, but combined deficiency was at least as frequent: concurrent iron and vitamin B_12_ deficiency was the single largest category (29.0%), exceeding isolated iron (24.4%) or isolated vitamin B_12_ deficiency (23.2%). Among anaemic children with all three analytes measured, 5841 (49.9%) had two or more concurrent deficiencies.

**Table 4:** Co-occurrence of iron, vitamin B_12_ and folate deficiency and associated red-cell size, among children with all three micronutrients measured (n=119 139)

Deficiency pattern	Children, n (%)	Anaemia within group, %	MCV median (IQR), fL	Normal MCV (≥80 fL), %
No deficiency	26 695 (22.4)	3.6	83.0 (80.0–86.2)	75.0
Iron	29 110 (24.4)	13.6	80.8 (77.3–84.1)	57.1
B_12_	27 602 (23.2)	3.4	84.8 (81.7–87.8)	84.2
Folate	184 (0.2)	6.0	85.4 (81.3–88.4)	84.2
Iron+B_12_	34 607 (29.0)	16.4	82.4 (78.5–85.7)	67.0
Iron+folate	220 (0.2)	24.1	81.8 (77.1–85.7)	61.4
B_12_+folate	306 (0.3)	2.9	87.1 (84.1–90.2)	92.8
Iron+B_12_+folate	415 (0.3)	23.4	83.9 (79.2–87.9)	70.8

Iron deficiency: ferritin <15 µg/L or transferrin saturation <16%; vitamin B_12_ deficiency <200 pg/mL; folate deficiency <3 ng/mL. Combined-deficiency rows are shown in bold. Normal MCV is defined as ≥80 fL*.*

MCV, mean corpuscular volume.

Combined deficiency had a direct bearing on the interpretation of red-cell indices. Among anaemic children with a normal MCV, 92.3% had at least one micronutrient deficiency and 53.7% had two or more, a proportion that slightly exceeded that seen in microcytic anaemia. Concordantly, the median MCV in anaemic children with two or more deficiencies was 74.6 fL, yet 24.8% of them fell within the normal range. Reliance on microcytosis alone as a trigger for micronutrient work-up would therefore have missed roughly one in four anaemic children with combined deficiency.

## Discussion

In this large, 7-year primary-care study of school-age children and adolescents, the prevalence of anaemia was 9.5% and was most strongly and independently associated with iron deficiency, alongside female sex and older age. The overwhelmingly microcytic morphology of anaemic children and the dominant contribution of iron deficiency delineate a clear, actionable picture: anaemia in this population is predominantly an iron-deficiency phenotype and is therefore largely preventable.^1 4^

Beyond confirming the expected iron-deficiency phenotype at unusually high precision, the principal contribution of this study lies in what the joint measurement of three micronutrients reveals. In a single provincial population, we show that combined deficiency is not a marginal phenomenon but the most common deficiency pattern among tested children, that concurrent iron and vitamin B_12_ deficiency exceeds either deficiency in isolation, and that a substantial share of combined-deficiency anaemia presents with a normal MCV. This last observation has a concrete clinical implication that a prevalence estimate alone cannot provide: red-cell size is an unreliable gatekeeper for micronutrient testing in this age group, and a normal MCV does not exclude treatable combined deficiency. To our knowledge, co-occurrence and morphology have not previously been described together at this scale in a Turkish primary-care population.

A prevalence of 9.5% represents a mild-to-moderate public-health problem and lies well below the global all-age estimate of 24.3%, consistent with the lower burden of middle-income and higher-income settings.^1 2 17^ It is almost identical to a recent, similarly designed Turkish primary-care study of children aged 6–18 years (9.4%), which supports the external validity of our estimate, while our substantially larger, multi-year sample provides more precise and temporally resolved figures.^16^

The most striking feature was the sex-dependent and age-dependent distribution of anaemia. Prevalence remained low and essentially flat with age in boys but rose steeply in girls—from 5.0% at 6–11 years to 13.7% at 12–14 years and 19.1% at 15–18 years—producing a widening female excess through adolescence. This is biologically coherent: menarche and recurrent menstrual blood loss, superimposed on the heightened iron demand of the pubertal growth spurt and frequently inadequate dietary iron intake, place post-menarcheal girls at particularly high risk.^5 6 13^ These findings identify adolescent girls as the principal target for preventive action.^14^

The predominance of microcytosis among anaemic children (76.0%), combined with iron deficiency being the strongest correlate in univariable (OR 4.98) and multivariable (adjusted OR 4.15) analyses, provides internally consistent evidence that iron deficiency is the leading cause of anaemia in this setting, concordant with global data.^3 11 12^ A practical corollary is that microcytic anaemia in a child—especially an adolescent girl—can reasonably be presumed iron-related pending confirmation, supporting pragmatic, ferritin-anchored diagnostic pathways.

Vitamin B_12_ deficiency was highly prevalent (53.7%), echoing elevated rates reported where the intake of animal-source foods is limited.^9 10 15^ Yet, after adjustment, vitamin B_12_ deficiency was not positively associated with anaemia. Two explanations are plausible: anaemia here is overwhelmingly iron-driven, so the marginal contribution of vitamin B_12_ is small once iron status is accounted for; and a single serum B_12_ below 200 pg/mL is an imperfect marker of functional deficiency and, without confirmatory methylmalonic acid or holotranscobalamin testing, may overstate true deficiency. Because vitamin B_12_ deficiency can cause neurocognitive consequences independently of megaloblastic anaemia, its high frequency warrants attention as a concern in its own right.^7 9^ By contrast, folate deficiency was rare (0.8%), consistent with adequate folate status in comparable settings and contributing negligibly to anaemia.^10^ Three further points bear on the high measured frequency of vitamin B_12_ deficiency. First, a threshold of 200 pg/mL identifies low circulating concentrations rather than confirmed tissue deficiency, and in the absence of methylmalonic acid or holotranscobalamin, the figure is best read as biochemical low-B_12_ status. Second, samples were analysed on a single centralised platform under continuous quality control, which limits between-site variability, although we cannot exclude assay recalibration over the 7-year window and did not attempt to model it. Third, the very high frequency is nonetheless consistent with independent Turkish data, so it is unlikely to be a purely analytical artefact.

Several features of the data source shape how these figures should be read. Testing was performed on clinical indication rather than by systematic screening, so the children analysed are those in whom a clinician judged a blood count or micronutrient assay to be warranted. This group is plausibly enriched for symptoms, poor growth, dietary concern or a prior suspicion of deficiency, and the resulting estimates are best understood as the yield of testing in routine primary care rather than as community prevalence, which they are likely to overstate. The same consideration applies to the associations: if children with deficiency were preferentially tested when also anaemic, the strength of association could be inflated. The internal consistency of the findings—an overwhelmingly microcytic, iron-associated pattern with a coherent age-and-sex gradient—argues against gross distortion, but absolute frequencies should not be transported to the general school-age population without a representative survey.

The analytes were not measured uniformly: haemoglobin was available in all children, whereas folate was measured in fewer than half, reflecting its more selective use in practice. This differential testing means that deficiency-specific denominators represent tested subgroups and may themselves be selected; folate in particular was both rarely measured and rarely deficient, which is why it was retained in univariable analysis but not carried into the multivariable model. We report every denominator explicitly so that the basis of each estimate is transparent.

The study period encompassed the COVID-19 pandemic, during which primary-care attendance and routine testing were markedly disrupted.^18^ Anaemia prevalence was modestly higher during the pandemic period and declined thereafter; given the very large sample, period differences were statistically significant. However, these differences must be interpreted cautiously, as pandemic-related changes in who was tested and why could alter the measured prevalence independently of any true change; ascertainment and case-mix effects are likely to contribute, and causal inference about a pandemic effect is not warranted from these cross-sectional data.

### Strengths and limitations

The strengths of this study are its large, multiyear and population-representative primary-care sample, standardised WHO age/sex-specific case definitions, high-resolution characterisation of sex-specific, age-specific and time-specific patterns, and rigorous data-quality control with physiological plausibility limits and de-duplication to one record per child. Several limitations apply. First, because data derive from children who underwent testing, findings generalise to the tested population rather than the general community, and attendees may be selected for anaemia or deficiency by underlying complaints. Second, the retrospective design precludes information on diet, socioeconomic status, chronic disease, medication, menstrual history and inflammatory markers (eg, C reactive protein), so inflammation-adjusted ferritin could not be assessed. Third, ferritin, vitamin B12 and folate were not measured in all individuals, restricting those analyses to tested subgroups. Fourth, diagnoses relied on single measurements, without confirmatory repeat testing or work-up for haemoglobinopathies. Finally, the single-province setting may limit external validity. Several additional limitations follow from the routine data source. The presence of intercurrent infection or inflammation at sampling was unknown, so acute-phase elevation of ferritin may have led us to underestimate iron deficiency; C reactive protein was not consistently available for inflammation adjustment. Common inherited disorders that mimic iron-deficient indices, in particular beta-thalassaemia trait, could not be excluded and may account for part of the microcytosis. Vitamin B12 and folate deficiency were defined on single serum measurements without functional confirmation. Finally, clinically relevant determinants of both anaemia and micronutrient status—diet, socioeconomic position, obesity, chronic inflammatory disease, menstrual history and medication—were not captured and could not be adjusted for, so residual confounding of the observed associations cannot be excluded.

## Conclusions

Anaemia is a common and largely preventable problem among school-age children and adolescents in primary care. Iron deficiency is the principal correlate and adolescent girls are the highest-risk group. These findings support strengthening iron-status screening, dietary interventions and iron-supplementation programmes—particularly for adolescent girls—while the high frequency of vitamin B12 deficiency, despite its limited contribution to anaemia, should be regarded as an independent public-health priority. In addition, combined micronutrient deficiency was common and frequently normocytic, so a normal MCV should not be taken to exclude treatable deficiency in an anaemic child.

## Supplementary material

10.1136/bmjpo-2026-005163 online supplemental table 1

10.1136/bmjpo-2026-005163 online supplemental file 1

## Data Availability

Data are available on reasonable request.
