# Supplementary material for: Anaemia and its association with iron, vitamin B12 and folate deficiency in school-age children and adolescents: a cross-sectional study of primary-care laboratory records (2019–2025)
Source: BMJ Paediatr Open. 2026 Sep 2;10(1):e005163. doi: 10.1136/bmjpo-2026-005163 (PMC13548245; doi:10.1136/bmjpo-2026-005163)
Supplement: Supplementary file 1 [file bmjpo-2026-005163.reviewer_comments.pdf]

PEER REVIEW HISTORY

BMJ Paediatrics Open publishes all reviews undertaken for accepted manuscripts. Reviewers are asked to complete a checklist review form and are provided with free text boxes to elaborate on their assessment. These free text comments are reproduced below.

ARTICLE DETAILS

**TITLE (PROVISIONAL)**	Anaemia and its association with iron, vitamin B12 and folate deficiency in school-age children and adolescents: a cross-sectional study of primary-care laboratory records (2019-2025)
**AUTHORS**	Erdoğan, Kevser<br>Doğan Tiryaki, Hülya<br>Kurt, Osman<br>Şeker, Nefise<br>Küçükkelepçe, Osman

VERSION 1 – REVIEW

**REVIEWER NAME**	Babacan, Oğuzhan
**REVIEWER AFFILIATION**	
**REVIEWER CONFLICT OF INTEREST**	No Conflict of Interest
**AI DISCLOSURE**	No, I have not used AI
**DATE REVIEW RETURNED**	

**GENERAL COMMENTS**	Potential limitations of the study include the lack of information regarding the presence of infection at the time of sample collection, the failure to exclude hereditary conditions that are common in Türkiye, such as beta-thalassemia trait, and the diagnosis of vitamin B12 deficiency without measuring methylmalonic acid or holotranscobalamin levels.<br><br>We suggest the following:<br><br>Although 76% of the identified anaemia cases were microcytic, the proportion of patients with concurrent iron and vitamin B12 and/or folate deficiencies should also be reported.<br>The mean corpuscular volume (MCV) may remain within the normal range in patients with combined iron and vitamin B12 and/or folate deficiencies. Presenting these data and highlighting that MCV can be normal in patients with concurrent iron and vitamin B12 deficiency would enhance the clinical relevance and overall value of the study.

**REVIEWER NAME**	Akyol, Şefika
**REVIEWER AFFILIATION**	Medical Park Hospitals Group, Division Pediatric Hematology and Oncology
**REVIEWER CONFLICT OF INTEREST**	No Conflict of Interest
**AI DISCLOSURE**	No, I have not used AI
**DATE REVIEW RETURNED**	27-Jul-2026

GENERAL COMMENTS	1. The exceptionally large sample size is a major strength, but the scientific novelty remains limited. The inclusion of more than 240,000 children represents one of the largest paediatric datasets reported from Türkiye and provides precise prevalence estimates with excellent statistical power. However, despite the impressive sample size, the manuscript offers relatively limited scientific novelty. The principal findings—that anaemia is more common in adolescent girls and that iron deficiency is the predominant correlate of anaemia—are already well established in the existing literature and are largely expected. The manuscript does not clearly demonstrate what new biological, epidemiological, or clinical knowledge is generated beyond providing more precise prevalence estimates. The authors should more explicitly define the unique scientific contribution of this study and clarify why this dataset substantially advances current knowledge rather than simply confirming previous observations.<br>2. The manuscript repeatedly refers to the prevalence of anaemia; however, the study population does not represent the general paediatric population. Instead, the analysis includes only children who attended primary care and underwent laboratory testing, which is a selected subgroup likely enriched for underlying symptoms, chronic diseases, nutritional concerns, or physician suspicion of anaemia. Although this limitation is acknowledged briefly, its potential impact on both prevalence estimates and observed associations deserves a much more detailed discussion.<br>3, The reported prevalence of vitamin B12 deficiency (53.7%) is remarkably high and deserves considerably more discussion than currently provided. Methodological issues about this like cut-off level of vitamin B12, any assay changes or recalibrations between 2019 and 2025, and any functional biomarkers used or not should be clarified.<br>4. The regression model includes only sex, age, iron deficiency and vitamin B12 deficiency. However, numerous clinically relevant variables that may influence both anaemia and micronutrient status were unavailable or not incorporated into the analysis, including socioeconomic status, nutritional habits, chronic inflammatory diseases, menstruel history, obesity, medications etc..<br>5. The manuscript does not sufficiently explain its unique contribution to the existing literature. The key question that remains unanswered after reading the manuscript is: Apart from including more than 240,000 children, what fundamentally distinguishes this study from the numerous previous cross-sectional studies reporting anaemia prevalence in children?<br><br>Overall recommendation: This manuscript has several notable strengths, particularly its exceptionally large sample size, robust data cleaning procedures, and use of standardized laboratory definitions. Nevertheless, its scientific novelty is currently insufficiently demonstrated, and several important methodological concerns—including selection bias, interpretation of the vitamin B12 findings, residual confounding in the regression analysis, and limited discussion of the study's unique contribution—should be addressed before the manuscript is suitable for publication in BMJ Paediatrics Open.

VERSION 1 – AUTHOR RESPONSE

Reviewer 1

We thank the Reviewer for highlighting the clinical dimensions of the data.

Comment (a) — limitations of the biomarkers. *Infection/inflammation status at sampling was unknown; common inherited conditions such as beta-thalassaemia trait were not excluded; and vitamin B12 deficiency was defined without methylmalonic acid or holotranscobalamin.*

Response. We agree and have expanded the limitations accordingly. We note that unrecognised inflammation may cause us to underestimate iron deficiency through acute-phase ferritin elevation; that beta-thalassaemia trait could not be excluded and may explain part of the microcytosis; and that low serum B12 without functional confirmation reflects biochemical rather than confirmed tissue deficiency.

Changes made. Strengths and limitations (p.11): three limitations added (infection/CRP, beta-thalassaemia trait, functional B12 confirmation). Discussion (B12 paragraph, p.9): the interpretive limits of a single low B12 are also discussed.

Comment (b) — combined deficiency. *Although 76% of anaemia was microcytic, the proportion of patients with concurrent iron and vitamin B12 and/or folate deficiency should be reported.*

Response. We thank the Reviewer for this valuable suggestion, which prompted the most substantial addition to the paper. Among the 119,139 children with all three micronutrients measured, combined deficiency was common: concurrent iron and vitamin B12 deficiency was the single largest category (29.0%), and 5,841 (49.9%) of anaemic children had two or more concurrent deficiencies. These are now presented in a new Table 5 and Figure 4.

Changes made. Results (new subsection "Co-occurrence of deficiencies and red-cell size", p.7): full co-occurrence analysis added, with new Table 5 and Figure 4.

Comment (c) — normal MCV in combined deficiency. *In combined iron plus B12/folate deficiency the MCV may remain normal; presenting these data and emphasising this point would enhance clinical value.*

Response. This is an important point and the data support it strongly. Among anaemic children with a normal mean corpuscular volume, 92.3% had at least one deficiency and 53.7% had two or more; indeed, the proportion of normocytic anaemia carrying two or more deficiencies (53.9%) slightly exceeded that in microcytic anaemia. We now emphasise that a normal MCV does not exclude treatable combined deficiency and that red-cell size is an unreliable gatekeeper for micronutrient testing.

Changes made. Results (p.7) and Figure 4B: masked (normocytic) combined deficiency quantified. Discussion (novelty paragraph, p.8) and Conclusion (p.11): clinical implication emphasised.

Reviewer 2

Comment 1 & 5 — scientific novelty / contribution to the literature. *The sample is large, but the findings (higher anaemia in adolescent girls, iron deficiency as the main correlate) are already known. What new biological, epidemiological or clinical knowledge does the study add beyond a more precise prevalence estimate?*

Response. We take this central point seriously and have addressed it directly rather than by appeal to sample size. Beyond confirming the iron-deficiency phenotype, the study's specific contribution is what the joint measurement of three micronutrients reveals: that combined deficiency—most often iron with vitamin B12—is the commonest deficiency pattern, exceeding either deficiency in isolation, and that about half of combined-deficiency anaemia is normocytic. This yields a concrete, practice-relevant message that a prevalence figure cannot: red-cell size should not gate micronutrient testing in this age group. We have added a dedicated paragraph articulating this contribution.

Changes made. Discussion (new paragraph, p.8): explicit statement of contribution built on the new co-occurrence and morphology analysis (Table 5, Figure 4). "What this study adds" (p.2): new bullet added.

Comment 2 — selection bias and use of “prevalence”. *The population is not the general child population but a selected subgroup tested on clinical indication; the effect of this on the prevalence estimates and on the associations should be discussed in much greater depth.*

Response. We agree and have substantially expanded this discussion. We now state that the estimates are best read as the yield of testing in routine primary care rather than community prevalence, which they likely overstate; that the associations could be inflated if deficient children were preferentially tested when anaemic; and that absolute frequencies should not be generalised without a representative survey. We retain the term prevalence but frame it explicitly as testing-based throughout.

Changes made. Discussion (new paragraph on the data source, p.9): in-depth treatment of selection and its effect on both estimates and associations. Abstract and Conclusion wording aligned to the tested-population framing.

Comment 3 — high vitamin B12 deficiency (53.7%). *This figure is very high and should be discussed further, including the threshold used, possible assay changes over 2019–2025, and whether a functional biomarker was used.*

Response. We have added a focused discussion. The 200 pg/mL threshold identifies low circulating concentrations rather than confirmed tissue deficiency; samples were analysed on a single centralised platform under continuous quality control, though we cannot exclude recalibration over seven years and did not model it; and no functional marker (methylmalonic acid or holotranscobalamin) was available. We also note that the high frequency is consistent with independent Turkish data and so is unlikely to be a purely analytical artefact.

Changes made. Discussion (B12 paragraph, p.9): three points added on threshold, assay/quality control and functional confirmation.

Comment 4 — residual confounding. *The model includes only sex, age, iron and B12 status. Many determinants (socioeconomic status, diet, chronic inflammatory disease, menstrual history, obesity, medication) are not included.*

Response. We agree and now state this explicitly as a limitation. These determinants of both anaemia and micronutrient status were not captured in routine laboratory records and could not be adjusted for, so residual confounding of the observed associations cannot be excluded.

Changes made. Strengths and limitations (p.11): sentence added listing the unmeasured determinants and acknowledging residual confounding.

Additional changes

For consistency with the current institutional records, the ethics statement now cites the İnönü University Health Sciences Scientific Research Ethics Committee (approval no. 2025-113). The artificial-intelligence declaration has been revised to reflect that language editing only was assisted. No results changed as a consequence of these edits.

We hope the revised manuscript now meets the standards of the journal and thank the Editor and Reviewers again for their time and expertise.
