# Supplementary material for: Anaemia and its association with iron, vitamin B12 and folate deficiency in school-age children and adolescents: a cross-sectional study of primary-care laboratory records (2019–2025)
Source: BMJ Paediatr Open. 2026 Sep 2;10(1):e005163. doi: 10.1136/bmjpo-2026-005163 (PMC13548245; doi:10.1136/bmjpo-2026-005163)
Supplement: Supplementary file 2 [file bmjpo-10-1-s001.docx]

**Supplemental Table 1.** Prevalence of anaemia by sex, age group and pandemic period

| **Variable**  Sex | **Category** | **Anaemic/total** | **%** | **p value**  **<0.001** |
| --- | --- | --- | --- | --- |
|  | Girls | 18,510/135,776 | 13.6 |  |
| Age group | Boys | 4,375/104,790 | 4.2 | **<0.001** |
|  | 6–11 years | 3,697/76,769 | 4.8 |  |
|  | 12–14 years | 5,771/59,675 | 9.7 |  |
| Period | 15–18 years | 13,417/104,122 | 12.9 | **<0.001** |
|  | Pre-pandemic | 5,018/56,374 | 8.9 |  |
|  | Pandemic | 9,209/86,859 | 10.6 |  |
|  | Post-pandemic | 8,658/97,333 | 8.9 |  |

*Comparisons used the chi-square test.*
